# Mass spectrometrical analysis of recombinant human growth hormone (Genotropin^®^) reveals amino acid substitutions in 2% of the expressed protein

**DOI:** 10.1186/1477-5956-3-1

**Published:** 2005-02-11

**Authors:** Felix Hepner, Edina Cszasar, Elisabeth Roitinger, Gert Lubec

**Affiliations:** 1 Department of Pediatrics, Medical University of Vienna, Vienna, Austria; 2 Mass Spectrometry Unit, University of Vienna, Austria

## Abstract

**Background:**

The structural integrity of recombinant proteins is of critical importance to their application as clinical treatments. Recombinant growth hormone preparations have been examined by several methodologies. In this study recombinant human growth hormone (rhGH; Genotropin^®^), expressed in *E. coli K12*, was structurally analyzed by two-dimensional gel electrophoresis and MALDI-TOF-TOF, LC-MS and LC-MS/ MS sequencing of the resolved peptides.

**Results:**

Electrospray LC-MS analysis revealed one major protein with an average molecular mass of 22126.8 Da and some additional minor components. Electrospray LC-MS/MS evaluation of the enzymatically digested Genotropin^® ^sample resulted in the identification of amino acid substitutions at the residues M_14_, M_125_, and M_170_; di-methylation of K_70 _(or exchange to arginine); deamidation of N_149_, and N_152_, and oxidation of M_140_, M_125 _and M_170_. Peak area comparison of the modified and parental peptides indicates that these changes were present in ~2% of the recombinant preparation.

**Conclusion:**

Modifications of the recombinant human growth hormone may lead to structural or conformational changes, modification of antigenicity and development of antibody formation in treated subjects. Amino acid exchanges may be caused by differences between human and E. coli codon usage and/or unknown copy editing mechanisms. While deamidation and oxidation can be assigned to processing events, the mechanism for possible di-methylation of K_70 _remains unclear.

## Background

The structural integrity of recombinant products generated by prokaryotic and eukaryotic organisms is a major concern. Modifications such as amino acid sequence substitution/mutations of recombinant proteins may lead to pharmacological inactivation, autoimmune phenomena [1-3] and adverse effects [4,5]. Human growth hormone (hGH) replacement is a frequent therapeutic intervention [6,7]. Genetic changes in human growth hormone have been linked to biological inactivity and disease: Lewis et al (2004) reported that a growth hormone variant I_179__M_179_ showed decreased ability to activate the extracellular signal-regulated kinase pathway and Binder et al. (2002) described hGH deficiency due to mutations of the coding regions of the growth hormone-1 gene [8,9]. Zhu et al. (2002) reported a case of hGH R_183__H_183_. This single mutation causes autosomal dominant growth hormone deficiency type II by prolonged retention time of R_183__H_183_ aggregates into secretory granules [10].

However, although such changes can be detrimental, non functional sequence alteration induced by poor editing of recombinant proteins may act as a marker of growth hormone abuse in situations such as athlete doping. We therefore were highly interested in the homogeneity and structure of rhGH preparations.

Genotropin^® ^is expressed by *E. coli, strain K12*. It consists of a single polypeptide chain containing 191 amino acids and two disulfide bonds (C_53_-C_165_; C_182_-C_189_) [11] with a molecular mass of 22 124 Da – representing the most abundant growth hormone form in humans [12].

In humans two major hGH splicing variants have been described, a 22 kDa protein and a 20 kDa protein, that bind different sites at the growth hormone receptor and serve different biological activities [13,14].

The genetic origin of hGH is the hGH-N gene, located on the long arm of chromosome 17, in a 66-kbp cluster region closely related to four other genes: hGH-V, hCS-A, hCS-B and hCS-L. The hGH-N gene is expressed in both, pituitary and several nonpituitary sites [12], all other gene products are produced by placental syncytio-trophoblasts.

A series of posttranslational modifications of hGH have been described and range from N-glycosylation, acetylation, deamidation, oxidation at M_14 _and M_125 _to polymerisation [12,15-18].

As mentioned above, Genotropin^® ^is expressed by E. coli. Since the fidelity of hGH translation in E. coli cannot rely on copy editing [19,20], nor on correct codon usage [21-23], there is a large potential for sequence errors. That's why investigations of structural/sequential integrity, including amino acid exchanges/mutations, and post translational modifications of rhGH Genotropin^® ^is of particular interest to for modern medicine and pharmacotherapy.

The aim of the present study was to investigate the homogeneity of a commercial available rhGH, Genotropin^®^. This was achieved using two dimensional gel electrophoresis (2-DE), matrix-assisted laser desorption/ionisation mass spectrometry (MALDI-MS) followed by tandem mass spectrometry (MALDI-MS/MS) and liquid chromatography mass spectrometry (LC-MS) followed by tandem mass spectrometry (LC-MS/MS). These modern analytical tools provide definitive structural analysis independent of antibody availability and specificity.

## Results

### Two dimensional gel electrophoresis

Two dimensional gel electrophoresis (2-DE) of 1 mg Genotropin^® ^showed a multiple spot pattern with masses between 20 000 and 35 000 Da and pIs from 4.5 to 7.0. Several two dimensional (2D) gels with sample amounts of 0.5, 1, 2, 5, 10, 20, 50, 100, 200 and 500 g of Genotropin^® ^were performed. Decreasing protein load showed reduction of spot size and number and finally, limitation to two spots of 22000 Da with pI of 5.3 and 5.4.

Neither MALDI-TOF-TOF nor LC-MS analysis of picked gel spots indicated any modifications or isoforms in an amount, that would explain differences between the two spots.

### Electrospray LC-MS measurements of the Genotropin^® ^sample

Electrospray liquid chromatography – mass spectrometry (LC-MS) measurements of the intact Genotropin^® ^have shown that the main product was a molecule with an average molecular weight (MW) of 22126.8 Da. The manufacturers had determined the average MW of Genotropin^® ^to be 22124 Da.

**Figure 1:**
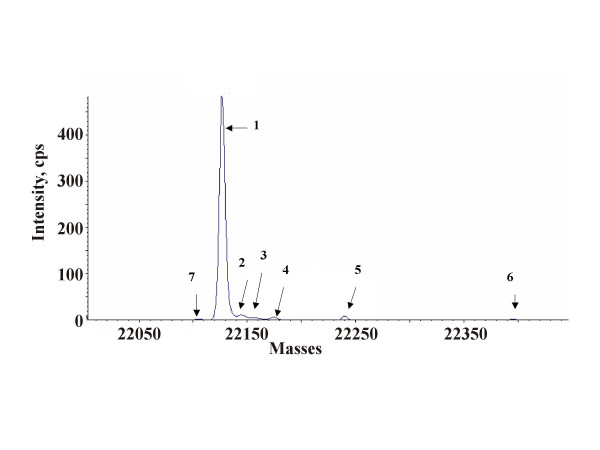
**Reconstructed electrospray LC/MS spectrum of the Genotropin^® ^sample. **The spectrum was recorded in positive ionization modus; 1 pM of the protein was injected. Detected average molecular masses: Nr.1.:22126.8 Da; Nr.2: 22143.5 Da; Nr.3: 22158.7 Da; Nr.4: 22174.4 Da; Nr.5: 22240.7 Da; Nr.6: 22395.9 Da; Nr7.: 22107.6 Da

The mass difference of approximately 3 Da may originate from the deconvulation of some broader, lower intensity peaks. Several minor components could be also detected. (Figure 1) The mass differences between the main product (Nr. 1) and components Nr. 2–5 respectively indicate the oxidation of several amino acid residues. Components Nr.6 and Nr.7 show a mass discrepancy of approximately +268 Da and -19 Da respectively. According to the ratios of peak areas (Table 1), the sample consists to 84.4% of the unmodified main component; the oxidation products are present to 13.7% of the whole sample and the ratio of other minor components, which may represent additional modifications or amino acid substitutions, is approximately 2%.

**Table 1:** Relative peak areas of the components detected by electrospray LC/MS measurement

**Molecular mass (Da)**	**Area (cps)**	**Area (%)**
22126.9	4909.8	84.4
22143.5	479.7	8.2
22158.7	121.5	2.1
22174.4	120.8	2.1
22107.6	76.9	1.3
22240.7	69.9	1.2
22395.9	40.4	0.7

Average molecular masses of the components detected in the LC/MS spectrum of 1 pM Genotropin^® ^sample. The peak areas were calculated from the reconstructed spectrum (Figure 1) recorded in positive ionization.

### Electrospray LC-MS/MS measurements following the tryptic digestion of the Genotropin^® ^sample

Electrospray tandem liquid chromatography – mass spectrometry (LC-MS/MS) measurements of the samples prepared from one dimensional SDS-PAGE indicated mass differences at several peptides. Doubly or triply charged ions were chosen for all MS/MS experiments due to their better fragmentation pattern. Table 2 shows the sequences of the modified peptides and possible explanations for the mass discrepancies.

**Table 2:** Results of electrospray LC-MS/MS and MALDI-TOF MS/MS measurements

**Sequence**	**M calculated (Da)**	**M observed (Da)**	**delta M (Da)**	**Modification**
EETQQ**K**SNLELLR	1586.83	1614.74	27.91	K_70_: di-methylation or → R *
FDT**N**SH**N**DDALLK	1488.68	1489.60	0.92	N_149 _/ N_152 _deamidation
**R**LEDGSPR	928.47	900.46	-28.01	R_127_→ Q or K *
LFDNA**M**LR	978.50	994.40	15.90	M_14 _: oxidation
D**M**DKVETFLR	1252.61	1268.40	15.79	M_170_: oxidation
DLEEGIQTL**M**GR	1360.61	1376.66	16.05	M_125_: oxidation
LFDNA**M**LR	978.50	960.40	-18.10	M_14 _→ I
DLEEGIQTL**M**GR	1360.61	1342.60	-18.01	M_125 _→ I
D**M**DKVETFLR	1252.61	1234.60	-18.01	M_170 _→ I
**L**FDNAMLR	978.50	1035.40	56.90	Carbamidomethyl – N terminus

• Nearly isobaric mass differences

Sequences and mass differences of modified peptides detected in the Genotropin sample. Column 1: sequence of modified peptides following tryptic digestion of Genotropin^®^; column 2: calculated monoisotopic masses of the unmodified tryptic peptides; column 3: observed monoisotopic masses; column 4: differences of calculated and observed masses; column 5: possible explanations of the mass differences.

**Figure 2:**
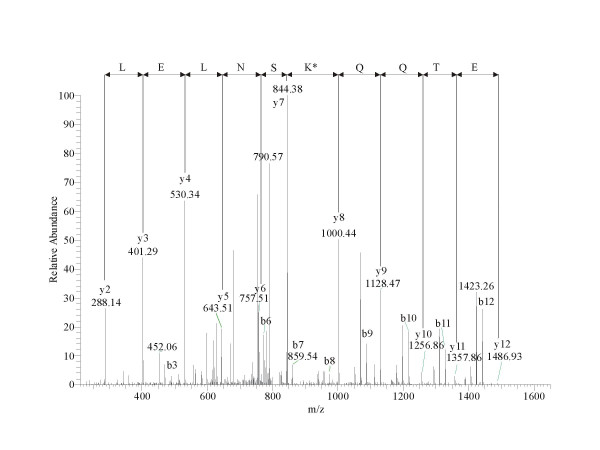
Electrospray LC-MS/MS spectrum of the modified peptide EETQQK*SNLELLR. Positive ionization product ion spectrum of the tryptic peptide m/z 808.4 generated by a linear ion trap mass spectrometer. Intensive y fragment ions verify that the K_170 _residue shows a mass increment of 28 Da compared to its theoretical mass.

A mass difference of +28 Da was detected at the position K_70 _(Figure 2), could be explained by the di-methylation of this residue, or by the exchange of this lysine to an arginine. These modifications result in a mass difference of 28.03 Da and 28.01 Da respectively. The accuracy of the mass spectrometric detection was not high enough to differentiate between these possibilities. Figure 2 shows the fragment spectrum of the peptide EETQQKSNLELLR. Intensive y ions verify that all residues have unchanged masses except of K_70_, which makes the localization of the mass discrepancy on that lysine residue unambiguous.

**Figure 3:**
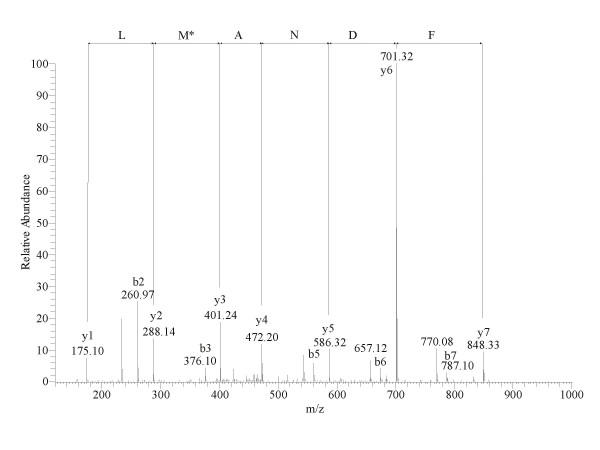
Electrospray LC-MS/MS spectrum of the peptide LFDNAM*LR. Positive ionization product ion spectrum of the tryptic peptide m/z 482.3 generated by a linear ion trap mass spectrometer. Ions of the y series verify the mass discrepancy of -18 Da at the M_14 _residue compared to its theoretical value.

Deamidation of the amino acids N_149 _and N_152 _was also detected. The molecular mass of the peptide RLEDGSPR was decreased with 28 Da. The mass difference could be localized to the N terminus of the peptide and might indicate the substitution of R_127 _with a lysine or glutamine. The mass difference of these residues is only 0.04 Da and the accuracy of the mass spectrometric detection was not high enough to differentiate between these amino acids. Residues M_14_, M_125 _and M_170 _were observed partly oxidized and in some cases the non oxidized residue showed a mass discrepancy of -18 Da (Table 2). This phenomenon is illustrated by Figure 3, which shows a product ion spectrum of the modified peptide LFDNAMLR. Fragment ions from the y series verify the mass reduction at the M_14 _residue. This mass difference can be explained by the replacement of these methionines with isoleucines, which can be originate from the substitution of the last base in the genetic codon of methionine (M:ATG; I:ATT/C/A). According to the ratios of the peak areas of the peptides containing the unmodified and possibly substituted methionines, these changes were present at < 2% of the whole protein amount. A mass increase of 57 Da was detected at the peptide LFDNAMLR. It could be localized at the N terminus of the peptide and it is supposed to be an artefact of the alkylation step during sample preparation. All modifications were partial; in each case peptides with both modified and unmodified residues were present. LC-MS/MS spectra for all modified peptides are available as supplementary material.

### MALDI analysis of Genotropin^®^

Approximately 96 spots were excised from a 2D gel with a sample load of 1 mg Gentotropin^® ^and identified by MALDI-TOF on the basis of peptide mass matching [24] following in gel digestion with trypsin. Those samples which were analysed by peptide mass fingerprinting from MALDI-TOF were additionally analysed using LIFT-TOF/TOF MS/MS from the same target. A maximum of three precursor ions per sample were chosen for MS/MS analysis.

**Figure 4:**
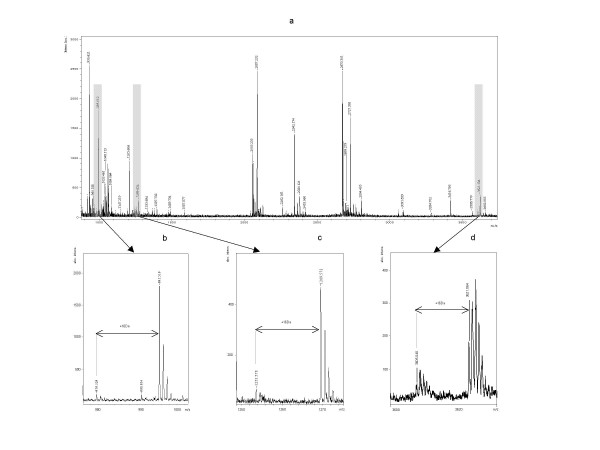
**MS spectrum of Genotropin showing oxidation of M_14_, M_125 _and M_170_.**(a) MS spectrum of Genotropin^® ^generated by an Ultraflex™ TOF/TOF (Bruker Daltonics) operated in the reflector mode for MALDI-TOF peptide mass fingerprint (PMF). Enlarged sections (b-d) from PMF of Genotropin^® ^showing oxidised/non-oxidised status (ÄM+16Da) of Methionine. (b) first peak: LFDNAMLR (979.529 Da)/ second peak: LFDNAMLR + oxidation of Methionine (995.519 Da); (c) first peak: DMDKVETFLR (1253.587 Da) / second peak: DMDKVETFLR + oxidation of Methionine (1269.572); (d) first peak: SVFANSLVYGASDSNVYDLLKDLEE-GIQTLMGR (3605.040) / second peak: SVFANSLVYGASDSNVYDLLKDLEE-GIQTLMGR + oxidation of Methionine (3621.064)

**Figure 5:**
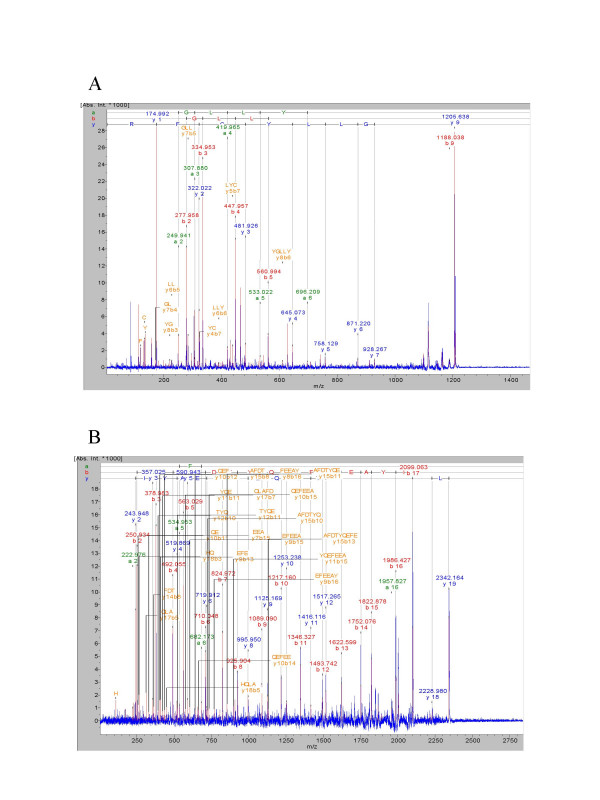
**MS/MS spectra of Genotropin^® ^.**LIFT-TOF/TOF (MS/MS) (a, b) spectra of Genotropin^® ^generated by an Ultraflex™ TOF/TOF (Bruker Daltonics) operated in LIFT mode for MALDI-TOF/TOF fully automated using the FlexControl™ software. The parent ions (m/z 1205.56 and m/z 2342.12) were selected for further analysis by MS/MS and the amino acid sequences NYGLLYCFR (5a) and LHQLAFDTYQEFEEAYIPK (5b) were unambiguously assigned to human growth hormone.

Genotropin^® ^was unambiguously identified by MS and MS/MS Data (Figure 4 and 5), with a maximum of 24 matching peptides, representing a sequence- coverage of 86% to human growth hormone sequence present in database (Figure 4, Table 3). All picked and analysed spots showed similar peptide mass fingerprints. Only the oxidation status of M varied, represented by a mass difference (ΔM) of 16 Da. Oxidation at M_14 _was demonstrated in 59,52% of analysed spots, 80,91% of M_125 _and 54,87% of M_170 _showed oxidation too (Table 3). Neither changes in amino acid sequence, nor post translational modifications like phosphorylation or deamidation could be detected by this method.

**Table 3:** Sequence coverage and peptide masses of Genotropin^® ^

Sequence Coverage: 86%						
Matched peptides shown in Bold						
1	**FPTIPLSRLF DNAMLRAHRL HQLAFDTYQE FEEAYIPKEQ KYSFLQNPQT**					
51	**SLCFSESIPT PSNREETQQK SNLELLRISL LLIQSWLEPV QFLRSVFANS**					
101	**LVYGASDSNV YDLLKDLEEG IQTLMGR**LED GSPRTGQIFK**QTYSKFDTNS**					
151	**HNDDALLKNY GLLYCFRKDM DKVETFLR**IV QCRSVEGSCG F					
Start – End	Observed	Mr(expt)	Mr(calc)	Delta	Miss	Sequence

1–8	930.61	929.60	929.53	0.07	0	FPTIPLSR
1–16	1906.97	1905.96	1906.01	- 0.05	1	FPTIPLSRLFDNAMLR Oxidation (M)
9–16	979.51	978.50	978.50	0.01	0	LFDNAMLR
17–38	2706.34	2705.33	2705.32	0.00	1	AHRLHQLAFDTYQEFEEAYIPK
20–38	2342.12	2341.11	2341.13	- 0.02	0	LHQLAFDTYQEFEEAYIPK
20–41	2727.35	2726.34	2726.32	0.02	1	LHQLAFDTYQEFEEAYIPKEQK
39–64	3058.51	3057.50	3057.45	0.05	1	EQKYSFLQNPQTSLCFSESIPTPSNR
42–64	2673.26	2672.26	2672.25	0.00	0	YSFLQNPQTSLCFSESIPTPSNR
42–70	3416.79	3415.78	3415.60	0.18	1	YSFLQNPQTSLCFSESIPTPSNREETQQK
65–77	1587.80	1586.79	1586.83	- 0.03	1	EETQQKSNLELLR
78–94	2055.19	2054.18	2054.19	- 0.01	0	ISLLLIQSWLEPVQFLR
95–115	2262.09	2261.08	2261.12	- 0.04	0	SVFANSLVYGASDSNVYDLLK
95–127	3621.02	3620.02	3619.77	0.25	1	SVFANSLVYGASDSNVYDLLKDLEEGIQTLMGR Oxidation (M)
116–127	1377.62	1376.62	1376.66	- 0.05	0	DLEEGIQTLMGR Oxidation (M)
141–158	2097.01	2096.00	2095.98	0.02	1	QTYSKFDTNSHNDDALLK
146–158	1489.66	1488.65	1488.68	- 0.03	0	FDTNSHNDDALLK
146–167	2676.26	2675.25	2675.24	0.01	1	FDTNSHNDDALLKNYGLLYCFR
159–167	1205.56	1204.55	1204.57	- 0.02	0	NYGLLYCFR
159–168	1333.64	1332.63	1332.66	- 0.03	1	NYGLLYCFRK
169–178	1253.58	1252.57	1252.61	- 0.04	1	DMDKVETFLR
169–178	1269.57	1268.56	1268.61	- 0.04	1	DMDKVETFLR Oxidation (M)

Sequence coverage of 86% and 21 matching peptides of Genotropin^® ^to human growth hormone could be detected using MALDI MS/ MS-MS data.

## Discussion

The dominant protein in the Genotropin^® ^preparation has an average molecular mass of 22127 Da. Electrospray LC-MS/MS evaluation of the trypsinized recombinant human growth hormone (rhGH) resulted in the identification of amino acid substitutions at residues M_14_, M_125 _and M_170_. Di-methylation of K_70 _or exchange to arginine, deamidation of N_149 _and N_152_, and oxidation of M_14_, M_125 _and M_170 _were also observed. These sequence alteration account for 2% of the recombinant protein.

Amino acid exchanges of a rhGH has been described before: Gellerfors et al. (1990) describe exchanges rhGH Q_65__V_65_ and rhGH Q_66__K_66_[25]. Since the product was not identified we cannot compare our results. Binding of recombinant human growth hormone to the GH receptor may be modified by the five amino acid exchanges observed in the present study. Pal et al. (2003) calculated binding energy differences between modified human growth hormone (hGHv; M_14__W_14_) and wild type human growth hormone (hGHwt; M_14_) with the result that the hGHv had more binding affinity to its receptor than hGHwt [26,27]. Cunningham et al. showed that M_14 _influenced binding, even if it is not a "hot spot" for linkage to its receptor. Furthermore, the amino acid exchanges detected may very well lead to antigenic differences and thus form the molecular basis for eliciting immune responses.

The underlying cause of amino acid exchanges may be codon usage and/or absence of copy editing in E. coli: The M_I exchanges may be due to miscast of the third nucleoside of the cognate anticodon at the so-called Wobble-position, i.e. switch cytosine to guanine/adenosine, a phenomenon described by Crick as "Wobble- hypothesis [28]. Crick (1966) postulated a certain amount of wobble at the third base position of the codon allowing more than one possible codon-anticodon- base pairing. Methionine (M)_I exchange of rhGH Genotropin^® ^may have been generated as the base pair G-G / G-A was replacing G-C. Arginine (R)_K/Q and R_G exchanges of rhGH Genotropin^® ^may be due to difficulties in translation of the rare codon AGG. Kane et al. (1995) predicted translational problems with an abundant mRNA species containing an excess of rare tRNA codons that may arise after the initiation of transcription of a cloned heterologous gene in the E. coli host [21]. Recent studies suggest clusters of AGG/AGA codons can reduce both quantity and quality of the synthesized protein [22,29]. Translational modification normally does not include amino acid exchanges but rather frameshift mutations/deletions [21,29-31].

In summary, we found two different pathways for amino acid exchanges in Genotropin^®^: translation errors due to usage of (1) the rare codon AGG in E. coli and (2) incorrect codon usage consisted with Crick's "Wobble-hypothesis".

Oxidative modification of a recombinant human growth hormone has been described by Karlsson et al. (1999) who demonstrated M_14 _and M_125 _oxidation as detected by LC-MS [32]. No other group have reported oxidation of M_170 _as in our study. Indeed Teh et al. (1987) oxidised natural hGH extracted from pituitary glands and detected M_14 _and M_125 _oxidation by reversed phase chromatography [33]. Gellerfors et al. (1990) oxidised rhGH with hydrogen peroxide but again failed to show oxidation of M_170_, as detected by reversed phase chromatography [25]. It is not known whether oxidation of methionines in recombinant human growth hormone leads to functional impairment but conformational changes are unlikely as proposed by circular dichroism and 1H-NMR studies [33]. It is worth mentioning that oxidised methionines are not localised at the receptor binding site.

Post translational modification such as N-acetylation, N-glycosylation, deamidation and oxidation have been reported for rhGH, hGH and bovine growth hormone (bGH) [15-17,34]. Dimethylation of K_70 _in rhGH and hGH have not previously been reported.

Whether transmethylation occurred during processing or is a post translational event during rhGH production in E. coli is unknown. Nevertheless, Martal et al. (1985) demonstrated reduction of biological activity of hGH and bGH by methylation and ethylation of its residues K_41_, K_70_, and K_115_[35]. Therefore, dimethylation of K_70 _in Genotropin^® ^could have biological relevance, probably reducing its pharmacotherapeutic activity.

Deamidation of N_149 _and N_152 _may be due to technical processing, probably by heat treatment or lyophilisation and has already been reported by Gellerfors et al (1990) and Karlsson et al. (1999) [25,32]. Though these appear to have no function significance [26,27,36].

Modifications of the recombinant human growth hormone, as shown in this study, may effect functionality and safety depending on the prevalence of such forms in the preparation. As already mentioned above, impaired binding to the receptor, conformational changes leading to impaired function, amino acid exchanges as mutations may well lead to immune phenomena or even disease [1-3,37]. In addition, such modifications may act as markers of these proteins in situations like rhGH doping.

## Conclusions

Using one- and two-dimensional gel electrophoresis, electrospray LC-MS, LC- MS/MS and MALDI-TOF-TOF mass spectrometry we detected a series of modifications of the recombinant human growth hormone (Genotropin^®^) including amino acid exchanges, oxidation, di-methylation and deamidation. This analytical battery is a reliable, specific and sensitive analytical tool for this purpose.

## Methods

### Sample preparation

Genotropin ^© ^MiniQuick 1,0 mg (Pharmacia & Upjohn; Stockholm, Sweden) was suspended in 0,5 ml of sample buffer consisting of 8 M urea (Merck, Darmstadt, Germany), 4% CHAPS (3- [(3-cholamidopropyl) dimethylammonio]-1-propane-sulfonate) (Sigma, St. Louis, MO, USA), 10 mM 1,4-dithioerythritol (Merck, Germany) and 0,5% carrier ampholytes "Resolyte" 3,5–10 (BDH Laboratory Supplies, Electran ^®^, England). The suspension was transferred into Ultrafree-4 centrifugal filter units (Millipore, Bedford, MA), for desalting and concentrating proteins. Protein content of the supernatant was quantified by the Bradford protein assay system [38]. The standard curve was generated using bovine serum albumin and absorbance was measured at 595 nm.

### One-dimensional SDS-polyacrylamide gel electrophoresis

One dimensional SDS-polyacrylamide gel was performed as described by Laemmli [39]. Samples of 0.5, 1, 2, 5, 10, 30, 50 and 100 μg were loaded on the gel. For determination of molecular weight 10 μl of precision plus protein standards, all blue (Bio Rad, California, USA), were applied on the gels.

### Two-dimensional gel electrophoresis (2-DE)

2 DE was performed essentially as reported [40]. Samples of 1 mg protein were applied on immobilized pH 3–10 nonlinear gradient strips in sample cups at their basic and acidic ends. Focusing was started at 200 V and the voltage was gradually increased to 8000 V at 4 V/min and then kept constant for a further 3 h (approximately 150,000 Vh totally). After the first dimension, strips (18 cm) were equilibrated for 15 min in the buffer containing 6 M urea, 20% glycerol, 2% SDS, 2% DTT and then for 15 min in the same buffer containing 2.5% iodoacetamide instead of DDT. After equilibration, strips were loaded on 9–16% gradient sodium dodecylsulfate polyacrylamide gels for second-dimensional separation. The gels (180 × 200 × 1.5 mm) were run at 40 mA per gel. Immediately after the second dimension run, gels were fixed for 12 h in 50% methanol, containing 10% acetic acid, the gels were stained with Colloidal Coomassie Blue (Novex, San Diego, CA) for 12 h on a rocking shaker. Molecular masses were determined by running standard protein markers (Biorad Laboratories, Hercules, CA) covering the range 10–250 kDa. pI values were used as given by the supplier of the immobilized pH gradient strips (Amersham Bioscience, Uppsala, Sweden). Excess of dye was washed out from the gels with distilled water and the gels were scanned with ImageScanner (Amersham Bioscience).

Electronic images of the gels were recorded using Adobe Photoshop and Microsoft Power Point Softwares.

### Matrix-assisted laser desorption ionisation mass spectrometry

Spots were excised with a spot picker (PROTEINEER sp™, Bruker Daltonics, Germany), placed into 96-well microtiter plates and in-gel digestion and sample preparation for MALDI analysis were performed by an automated procedure (PROTEINEER dp™, Bruker Daltonics) [41,42]. Briefly, spots were excised and washed with 10 mM ammonium bicarbonate and 50% acetonitrile in 10 mM ammonium bicarbonate. After washing, gel plugs were shrunk by addition of acetonitrile and dried by blowing out the liquid through the pierced well bottom. The dried gel pieces were reswollen with 40 ng/μl trypsin (Promega, U.S.A.) in enzyme buffer (consisting of 5 mM Octyl β-D-glucopyranoside (OGP) and 10 mM ammonium bicarbonate) and incubated for 4 hrs at 30°C. Peptide extraction was performed with 10 μl of 1% TFA in 5 mM OGP. Extracted peptides were directly applied onto a target (AnchorChip™, Bruker Daltonics) that was load with α-cyano-4-hydroxy-cinnamic acid (Bruker Daltonics) matrix thinlayer. The mass spectrometer used in this work was an Ultraflex™ TOF/TOF (Bruker Daltonics) operated in the reflector mode for MALDI-TOF peptide mass fingerprint (PMF) or LIFT mode for MALDI-TOF/TOF fully automated using the FlexControl™ software. An accelerating voltage of 25 kV was used for PMF. Calibration of the instrument was performed externally with [M+H]^+ ^ions of angiotensin I, angiotensin II, substance P, bombesin, and adrenocorticotropic hormones (clip 1–17 and clip 18–39). Each spectrum was produced by accumulating data from 200 consecutive laser shots. Those samples which were analysed by PMF from MALDI-TOF were additionally analysed using LIFT-TOF/TOF MS/MS from the same target. A maximum of three precursor ions per sample were chosen for MS/MS analysis. In the TOF1 stage, all ions were accelerated to 8 kV under conditions promoting metastable fragmentation. After selection of jointly migrating parent and fragment ions in a timed ion gate, ions were lifted by 19 kV to high potential energy in the LIFT cell. After further acceleration of the fragment ions in the second ion source, their masses could be simultaneously analysed in the reflector with high sensitivity. PMF and LIFT spectra were interpreted with the Mascot software (Matrix Science Ltd, London, UK). Database searches, through Mascot, using combined PMF and MS/MS datasets were performed via BioTools 2.2 software (Bruker). A mass tolerance of 100 ppm and 2 missing cleavage sites for PMF and MS/MS tolerance of 0.5 Da and 1 missing cleavage sites for MS/MS search were allowed and oxidation of methionine residues was considered. The probability score calculated by the software was used as criterion for correct identification.

The algorithm used for determining the probability of a false positive match with a given mass spectrum is described elsewhere [43].

### Nano-electrospray LC-MS and LC-MS/MS analysis

Genotropin^® ^MiniQuick 0.6 mg (Pharmacia & Upjohn; Stockholm, Sweden) was suspended in the solution provided in the two-chamber cartridge and diluted with 1% formic acid (Merck; Darmstadt, Germany) in water (Maxima, Elga; High Wycombe, UK) to 1 pM/μl. 1 μl of this solution was used for the nano-electrospray LC-MS investigation. The HPLC used was an UltiMate™ system (Dionex Corporation; Sunnyvale, CA, USA) equipped with a PepMap C18 purification column (300 μm × 5 mm) and a 75 μm × 150 mm analytical column of the same material. 0.1% TFA (Pierce Biotechnology Inc.; Rockford, IL, USA) was used on the Switchos module for the binding of the peptides and a linear gradient of acetonitrile (Chromasolv^®^, Sigma-Aldrich; Seelze, Germany) and 0.1% formic acid in water was used for the elution. The gradient was (A = 5% acetonitrile / 0.1% formic acid in water; B = 80% acetonitrile / 0.1% formic acid in water) 0% B for 12 min, 80% B in 30 min, 100 % B in 3 min, 100% B for 10 min, 0% B in 2 min, 0% B for 23 min. The flow rate was 240 nl/min. The LC-system was coupled on-line to a QSTAR Pulsar hybrid mass spectrometer (Applied Biosystems; Foster City, CA, USA). The nanospray source of Proxeon (Odense, Denmark) was used with the distal coated silica nanospray capillaries of New Objective (Woburn, MA, USA). The electrospray voltage was set to 1800 V. Spectra were acquired over the mass range of m/z 600–1600. The accumulation time was 1 sec. Protein spectra were deconvoluted by Analyst^® ^(Applied Biosystems; Foster City, CA, USA). LC-MS/MS analyses were carried out also with the UltiMate™ system interfaced to the QSTAR Pulsar or to an LTQ (Thermo; San Jose, CA, USA) linear ion trap mass spectrometer. The gradient was (A = 5% acetonitrile / 0.1% formic acid in water B = 80% acetonitrile / 0.1% formic acid in water) 0% B for 12 min, 60% B in 88 min, 100 % B in 5 min, 100% B for 10 min, 0% B in 5 min, 0% B for 20 min. Peptide spectra were recorded over the mass range of m/z 450–1300, MS/MS spectra were recorded in information dependent data acquisition over the mass range of m/z 50–1600. One peptide spectrum was recorded followed by two MS/MS spectra on the QSTAR Pulsar instrument; the accumulation time was 1 sec for peptide spectra and 2 sec for MS/MS spectra. The collision energy was set automatically according to the mass and charge state of the peptides chosen for fragmentation. One full spectrum was recorded followed by 3 MS/MS spectra on the LTQ instrument, automatic gain control was applied and the collision energy was set to the arbitrary value of 35. Doubly or triply charged ions were selected for product ion spectra. MS/MS spectra were interpreted by Mascot (Matrix Science Ltd, London, UK).

## Competing interests

The author(s) declare that they have no competing interests.

## Authors' contributions

Felizardo, Maureen carried out MALDI-TOF-TOF analysis. Raja, Karlin participated in sequence alignments and in the design of the study. All authors read and approved the final manuscript.
